# N-Halamine-Based Polypropylene Melt-Blown Nonwoven Fabric with Superhydrophilicity and Antibacterial Properties for Face Masks

**DOI:** 10.3390/polym15214335

**Published:** 2023-11-06

**Authors:** Zhuo Chen, Qinghua Zhao, Jiahui Chen, Tao Mei, Wenwen Wang, Mufang Li, Dong Wang

**Affiliations:** 1 Key Laboratory of Textile Fiber and Products, Wuhan Textile University, Ministry of Education, Wuhan 430200, China; chenzhuo9@126.com (Z.C.); zhaoqh108@126.com (Q.Z.); meitao006@foxmail.com (T.M.); wwang@wtu.edu.cn (W.W.); wangdon08@126.com (D.W.); 2 College of Textile Science and Engineering, Wuhan Textile University, Wuhan 430200, China

**Keywords:** polypropylene melt-blown nonwoven fabric, antibacterial materials, UV irradiation, superhydrophilicity, N-halamine

## Abstract

Polypropylene melt-blown nonwoven fabric (PP MNF) masks can effectively block pathogens in the environment from entering the human body. However, the adhesion of surviving pathogens to masks poses a risk of human infection. Thus, embedding safe and efficient antibacterial materials is the key to solving pathogen infection. In this study, stable chlorinated poly(methacrylamide-N,N′-methylenebisacrylamide) polypropylene melt-blown nonwoven fabrics (PP-P(MAA-MBAA)-Cl MNFs) have been fabricated by a simple UV cross-link and chlorination process, and the active chlorine content can reach 3500 ppm. The PP-P(MAA-MBAA)-Cl MNFs show excellent hydrophilic and antibacterial properties. The PP-P(MAA-MBAA)-Cl MNFs could kill all bacteria (both *Escherichia coli* and *Staphylococcus aureus*) with only 5 min of contact. Therefore, incorporating PP-P(MAA-MBAA)-Cl MNF as a hydrophilic antimicrobial layer into a four-layer PP-based mask holds great potential for enhancing protection and comfort.

## 1. Introduction

In recent years, the harmful impact of airborne pathogens on human health and development has become increasingly evident [1,2,3,4,5,6]. With the densification of the world’s population and the acceleration of globalization, the outbreak and spread of infectious diseases caused by airborne pathogens have become increasingly prominent. These pathogens can be suspended in the air in the form of tiny aerosol particles and inhaled through the respiratory tract, causing disease transmission [7,8]. The three-dimensional disordered fiber structure of polypropylene-based medical protective masks and the high-efficiency filtration characteristics of electrostatic electrets can effectively intercept droplets or aerosols in the inhaled air, which is the first measure for people to resist the invasion of airborne pathogens [9,10,11]. In protective masks, PP MNFs are commonly utilized as the core layer, providing essential filtration functionality, and are typically laminated with support materials to create multi-layer composite materials [12]. For example, ordinary medical protective masks generally contain a three-layer structure consisting of polypropylene spun-bond nonwoven fabric (S), polypropylene melt-blown nonwoven fabric (M), and polypropylene spun-bond nonwoven fabric (S)—namely, an SMS structure. The M layer can also be superimposed to form an SMMS or SMMMS structure to achieve a higher filtering effect [13]. However, when individuals wear masks, whether inhaling or exhaling, the mask captures pathogens dispersed by the environment or the human body. As PP MNFs lack the capacity to deactivate pathogens, the surviving pathogens can persist in the material in the absence of external interference, which presents a significant risk for secondary infections [14]. Therefore, endowing PP MNFs with the ability to inactivate pathogens will significantly eliminate the potential infection risk present in PP MNF masks.

To solve the problem of pathogen residue, various antibacterial modification techniques of polypropylene melt-blown materials have been widely studied, such as combining metals or metal oxides [15,16], graphene or graphene oxide [17,18,19], bio-based materials [20], metal-organic frameworks [21,22], N-halamine [23,24], quaternary ammonium salt [25], etc. Among various antibacterial agents, N-halamine possesses several desirable characteristics, including a broad-spectrum antibacterial effect, excellent physical and chemical stability, reproducible antibacterial performance, and safety for the human body and the environment [26,27,28,29,30,31]. In light of these properties, embedding N-halamine into PP MNFs represents a safe and effective solution for producing medical materials with antibacterial properties. This development holds immense potential for application in medical protection. Ma’s group [23] successfully developed a high-performance polypropylene-grafted methacrylamide nonwovens (PP-*g*-MAM nonwovens) through reactive extrusion and melt-blown processes. After chlorination, the active chlorine content was >850 ppm, showing the inactivation effect on various microorganisms. However, in the PP-*g*-MAM nonwovens prepared by the melt-blown process, only the N-halamine groups exposed on the fiber surface can be chlorinated, leading to the inability to fully utilize the N-halamine grafted on the polypropylene molecular chain. Moreover, when attempting to graft N-halamine onto polypropylene molecular chains using free radical melt grafting, the inherent inertness of polypropylene presents a great process challenge.

Currently, the vast potential of nano-sized N-halamine in contact sterilization stems from its small size and high specific surface area [32,33]. Gutman’s research [34] reported a novel nanoparticle based on N-halamine. The new nanoparticles (P(MAA-MBAA)) were prepared via the surfactant-free dispersion co-polymerization of methacrylamide (MAA) and N,N-methylenebisacrylamide (MBAA) in a continuous aqueous phase with potassium persulfate as the initiator. The novel nanoparticles are relatively easy to synthesize and showed excellent antimicrobial activity and recharging capacity. In investigating the potential application of P(MAA-MBAA) nanoparticles, Natan et al. [35] incorporated a physical blend of P(MAA-MBAA) nanoparticles and polyethylene powder into the injection molding machine to manufacture drippers. Functionalized drippers have been shown to effectively resist biofilm formation, maintaining anti-fouling activity for at least five months when used in wastewater irrigation, and their anti-fouling performance can be extended through re-chlorination. However, only the nanoparticles located on the surface of the drippers exhibit anti-fouling functionality, while those blended within the drippers have negligible effects, which greatly reduces the usability of the nanoparticles and limits the increase in active chlorine content on functionalized drippers.

To tackle the challenges associated with the hard grafting of polypropylene and the limited utilization of N-halamine, a rapid and facile approach was adopted to generate a coating consisting of P(MAA-MBAA) nanoparticles on the surface of PP MNFs in this study. Notably, a significant number of P(MAA-MBAA) nanoparticles were successfully generated on the PP MNF fibers after a brief period of UV treatment, exhibiting a homogenous distribution across the entire surface of the PP MNFs. The resulting PP-P(MAA-MBAA)-Cl MNFs produced after chlorination showed excellent antibacterial properties, high active chlorine content, super hydrophilicity, and a rechargeable function. The effects of monomer, cross-linker, photoinitiator, and UV irradiation time on P(MAA-MBAA) nanoparticle content in PP MNFs were thoroughly investigated. After chlorination treatment, the resulting PP-P(MAA-MBAA)-Cl MNFs displayed highly active chlorine content and demonstrated rapid and effective bacterial killing. Furthermore, incorporating P(MAA-MBAA) nanoparticles significantly enhanced the hydrophilicity of PP MNFs and facilitated the efficient transport of external moisture. These findings highlight the potential of PP-P(MAA-MBAA)-Cl MNFs as highly effective and versatile materials for various antibacterial and healthcare applications.

## 2. Materials and Methods

### 2.1. Materials

Polypropylene melt-blown nonwoven fabrics (the areal density of the fabrics is 24 GSM) and polypropylene spun-bonded nonwoven fabrics (the areal density of the fabrics is 26 GSM) were purchased from Tuoying New Material, Hubei, China. Methacrylamide (MAA), N, N′-methylenebisacrylamide (MBAA) and benzophenone (BP) were purchased from Aladdin, China. Potassium iodide (KI), soluble starch, sulfuric acid, sodium hypochlorite (NaOCl, CP, active chlorine ≥ 5.2%), anhydrous ethanol, and acetone were purchased from Sinopharm, China. Sodium thiosulfate solution (Na_2_S_2_O_3_, 1.000 mol/L) was purchased from RHAWN, China. *Escherichia coli* (ATCC 25922) and *Staphylococcus aureus* (ATCC 2913) were purchased from Lezhen, Jiangsu, China.

### 2.2. Preparation of PP-P(MAA-MBAA)-Cl MNFs

**Figure 1:**
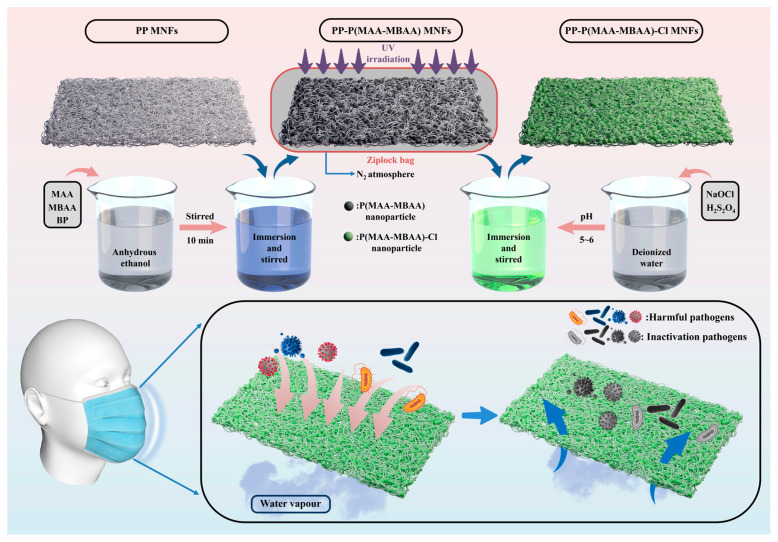
Schematic illustration of the preparation process of PP-P(MAA-MBAA) MNFs.

The preparation of PP-P(MAA-MBAA)-Cl MNFs is shown in Figure 1. First, the PP MNFs (6 × 6 cm) were added into the solution (15 wt% MAA, 3 wt% MBAA, and 1 wt% BP) and shaken for 10 min at room temperature. Subsequently, the impregnated PP MNFs were placed in a polyethylene Ziplock bag with a nitrogen atmosphere and put into an ultraviolet light box. The ultraviolet light box consisted of an opaque container, an ultraviolet lamp (120 W power, 10 cm from the bottom), and heat dissipation fans. After the end of UV treatment, unreacted monomers in PP-P(MAA-MBAA)-Cl MNFs were removed with excess absolute ethanol and deionized water and then dried at 60 °C. Finally, the chlorination treatment was completed by adding PP-P(MAA-MBAA) MNFs to aqueous sodium hypochlorite (15 mL NaOCl with 100 mL water to dilute, adjust pH between 5 and 6) to obtain PP-P(MAA-MBAA)-Cl MNFs. The active chlorine content loaded on the PP-P(MAA-MBAA)-Cl MNFs was determined by iodometric/thiosulfate titration (the chemical reaction of each treatment step can be seen in Figure S1).

### 2.3. Characterization of Physicochemical Properties

Fourier transform infrared (FTIR) spectroscopy (Bruker, VERTEX70, Billerica, MA, USA) was recorded over the wavelength range of 400–4000 cm^−1^. The surface micromorphology of the PP MNFs and the PP-P(MAA-MBAA) MNFs was observed using a scanning electron microscope (JSM-IT300A, Tokyo, Japan). X-ray photoelectron spectroscopy (Thermo Scientific) and energy dispersive spectroscopy (JEOL-6510, Tokyo, Japan) were used to examine the surface elemental composition and elemental mapping images, respectively. The mechanical behavior and dynamic contact angle were characterized by an electronic tensile machine (INSTRON 5967, Norwood, MA, USA) and contact-angle measurement (Kruss, DSA30S, Hamburg, Germany). The filtration efficiency and air permeability of the PP MNFs and the PP-P(MAA-MBAA) MNFs were tested using a filter material comprehensive performance test bench (LZC-H, Suzhou, China) and a fabric air permeability tester (YG(B) 461G, Dahe, Ningbo, China), respectively.

### 2.4. Antibacterial Assays against Escherichia coli and Staphylococcus aureus

The antibacterial activity of the PP-P(MAA-MBAA)-Cl MNFs was tested against *Escherichia coli* and *Staphylococcus aureus*. The bacteria were cultured using the liquid culture method, and the second generation of bacteria was used for the antibacterial test. The cultured bacteria were diluted, centrifuged with PBS buffer, and prepared as a bacterial suspension with a 10^6^ CFU/mL concentration. The unprocessed bacterial suspension was used as the control group, and the PP-P(MAA-MBAA)-Cl MNFs sample (0.15 g) was used as the experimental group. We added the PP-P(MAA-MBAA)-Cl MNF sample to 5 mL of 10^6^ CFU/mL bacterial suspension. After 0, 5, 15, 30, and 60 min of contact between the PP-P(MAA-MBAA)-Cl MNFs and bacterial suspension, 0.5 mL of the bacterial suspension was taken and diluted 100 times, respectively. The diluted bacterial suspension was spread on an agar plate using a spreader, and the agar plates were incubated in a 37 °C incubator for 24 h. The antibacterial effect of the sample was observed after the bacteria grew and formed colonies.

To further simulate the real scene, a *Staphylococcus aureus* suspension with a concentration of 10^6^ CFU/mL was added to the nebulizer for aerosolization, aiming to mimic the aerosol state containing bacteria in a real environment. In the experiment, the PP-P(MAA-MBAA)-Cl MNFs were placed at the nozzle of the nebulizer for 10 s. Subsequently, the PP-P(MAA-MBAA)-Cl MNFs carrying the bacterial aerosol were placed in a culture box for 24 h to allow contact between the bacteria and the PP-P(MAA-MBAA)-Cl MNFs. Afterward, the PP-P(MAA-MBAA)-Cl MNFs were transferred to agar plates and incubated for 24 h to observe the antibacterial effect of the PP-P(MAA-MBAA)-Cl MNFs on the bacteria.

### 2.5. Active Chlorine Content

After the end of UV treatment, unreacted monomers in the PP-P(MAA-MBAA)-Cl MNFs were removed with excess absolute ethanol and deionized water and then dried at 60 °C. Finally, chlorination treatment was completed by adding PP-P(MAA-MBAA) MNFs to aqueous sodium hypochlorite (15 mL NaOCl with 100 mL water to dilute, adjust pH between 5 and 6) to obtain PP-P(MAA-MBAA)-Cl MNFs. The active chlorine content loaded on the PP-P(MAA-MBAA)-Cl MNFs was determined using iodometric/thiosulfate titration [36]. The following formula calculates the active chlorine content [37]:

$$Active\;Chlorine\;Content\left(ppm\right)=\frac{35.45\times N\times V\times 1000}{2\times W}$$

where *N* is the normality (equiv./L) of sodium thiosulfate solution, *V* refers to the volumes (mL) of sodium thiosulfate solution consumed in the titration process, and *W* (g) is the dry weight of the PP-P(MAA-MBAA)-Cl MNFs.

## 3. Results and Discussion

The strong oxidation state of the halogen atom in the N-X bond makes the halamine antibacterial agent exhibit strong antibacterial activity against pathogens [26]. With the continuous improvement of people’s pursuit of healthy life, the harmful effects of pathogens in the air on human health and development are becoming more and more obvious. Therefore, it is very important to apply N-halamine to filter materials and inactivate pathogens blocked by the surface of masks. We propose a simple method to obtain a mask with a high antibacterial effect, as shown in Figure 1, and the specific method is as follows: PP MNFs were added into the solution and shaken for 10 min at room temperature to fully attach the N-halamine monomer. PP-P(MAA-MBAA) MNFs were obtained using an ultraviolet lamp cross-linked in a polyethylene Ziplock bag with a nitrogen atmosphere (Figure S2). After chlorination using aqueous sodium hypochlorite, active chlorine content can reach 3500 ppm. Because of the excellent antibacterial properties of PP-P(MAA-MBAA)-Cl MNFs, the pathogens on the mask can be inactivated, thereby preventing secondary infection.

**Figure 2:**
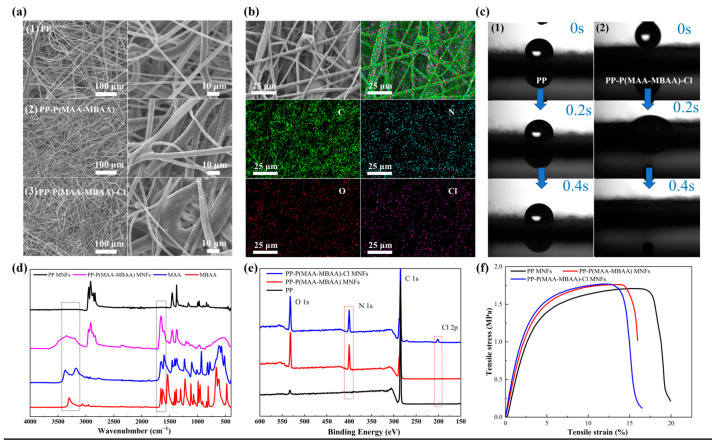
(**a**) Microstructure of PP, PP-P(MAA-MBAA) and PP-P(MAA-MBAA)-Cl MNFs. (**b**) EDS mapping image of C, N, O, Cl distribution in the PP-P(MAA-MBAA)-Cl MNFs. (**c**) The water contact angle of PP and PP-P(MAA-MBAA)-Cl MNFs. (**d**) FTIR spectra of the PP MNFs, MAA, MBAA, PP-P(MAA-MBAA) MNFs. (**e**) Full scan of the XPS spectra of PP-P(MAA-MBAA)-Cl, PP-P(MAA-MBAA), and PP MNFs. (**f**) Mechanical properties of PP, PP-P(MAA-MBAA), and PP-P(MAA-MBAA)-Cl MNFs.

The homogeneous distribution of P(MAA-MBAA) nanoparticles on the fibers is crucial for achieving comprehensive antibacterial effectiveness in PP MNFs, which will result in uniform antimicrobial properties. The surface morphologies of the PP-P(MAA-MBAA) MNFs were characterized by SEM and are illustrated in Figure 2a. The P(MAA-MBAA) nanoparticles were evenly dispersed as scattered points on the PP MNFs’ surface. The formation process of P(MAA-MBAA) nanoparticles is as follows: sufficient reactive monomers were attached to the PP MNFs after solution treatment, and then, under UV irradiation, benzophenone can absorb energy to induce the polymerization of methylacrylamide and N,N-methylenebisacrylamide. During the reaction, the carbonyl carbon atom in the acrylamide group combines with the propenyl carbon atom in the methylene acrylamide molecule to form a comonomer group. When the reaction proceeds, not only polymerization between monomers occurs, but a cross-linked network is also formed. This means that the molecules inside the nanoparticles connect to each other to form a stable three-dimensional structure. As the reaction proceeds, the cross-linked network gradually expands, eventually forming P(MAA-MBAA) nanoparticles. As the number of nanoparticles increases, a coating composed of P(MAA-MBAA) nanoparticles gradually forms on the surface of the PP MNF fiber. P(MAA-MBAA) nanoparticles had a lot of N-halamine and polar hydrophilic groups, which could provide high active chlorine content and an excellent hydrophilic effect for killing pathogens and water transport. After chlorination, P(MAA-MBAA) nanoparticles remain firmly bound to the fiber surface (Figure 2(a2). Furthermore, energy dispersive X-ray spectroscopy (EDS) analysis (Figure 2b) revealed the presence of Cl elements on the surface of PP-P(MAA-MBAA)-Cl MNFs, which indicates that the −NH_2_ in P(MAA-MBAA) nanoparticles converts into −NHCl to realize PP MNFs with antibacterial function. Therefore, P(MAA-MBAA) nanoparticles were coated on PP MNFs after simple processing, and the PP-P(MAA-MBAA)-Cl MNFs were obtained after chlorinating PP-P(MAA-MBAA) MNFs. Notably, the P(MAA-MBAA) nanoparticles exhibit super hydrophilicity. As shown in Figure 2c, because of the non-polar structure, the PP MNFs surface cannot be wetted by the water drop, and the water contact angles are as high as 142°. However, once the water drop comes into contact with the surface of the PP-P(MAA-MBAA) MNFs, it can quickly spread and be absorbed. The PP-P(MAA-MBAA) MNFs of super hydrophilicity can be added into the protective mask as a hydrophilic functional layer, which is used to absorb droplets formed by human exhaled moisture to reduce the discomfort of the long-term wearing of the polypropylene-based mask. Figure 2d shows the FTIR spectra of the MAA, MBAA, PP MNFs, and PP-P(MAA-MBAA) MNFs. Compared with the PP MNFs, the new peaks at 1660 and 3352 cm^−1^ appearing on the spectrum of the PP-P(MAA-MBAA) MNFs are attributed to the vibrations of C=O (amide I stretch) and N–H (symmetric vibration) from the amide group, respectively, indicating that the N-halamine was introduced onto the surface of PP MNFs. Moreover, compared with the spectrum of MAA, disappear of the C=C bond at 1603 and the C−H bonds at 933 cm^−1^ in P(MAA-MBAA) nanoparticles, implying that the P(MAA-MBAA) nanoparticles do not contain unreacted monomers, which is similar to the results of Gutman’s study [34]. In the XPS spectra (Figure 2e), a strong peak at 399 eV, corresponding to the binding energy of N 1s [38], was observed in the PP-P(MAA-MBAA) MNFs. After chlorination, the PP-P(MAA-MBAA)-Cl MNFs showed significant enhancement at the binding energy of 202 eV, corresponding to the binding energy of Cl 2p, which indicates that the N-H bond in the P(MAA-MBAA) nanoparticles has been transformed into an N-Cl bond [39]. The release of the active chlorine in the N-Cl bond provides an effective guarantee for the antibacterial function of PP nonwovens.

The PP-P(MAA-MBAA)-Cl MNFs have excellent mechanical properties, as shown in Figure 2f. Compared with the PP MNFs, the PP-P(MAA-MBAA) MNFs demonstrate a slight improvement in tensile strength, reaching 1.76 MPa. This enhancement may be attributed to the aggregation of certain P(MAA-MBAA) nanoparticles at the fiber bonding sites, resulting in a strengthened inter-fiber connection. However, the PP-P(MAA-MBAA) MNFs exhibited a decrease in fracture elongation rate, which could be attributed to an increase in material compactness after multiple solvent treatments, thereby limiting fiber extensibility. The decrease in fracture elongation rate also occurred in PP-P(MAA-MBAA)-Cl MNFs treated with chloride solution, indicating a limit to the times of solution treatments that can be applied without excessively affecting the mechanical properties of PP MNFs.

**Figure 3:**
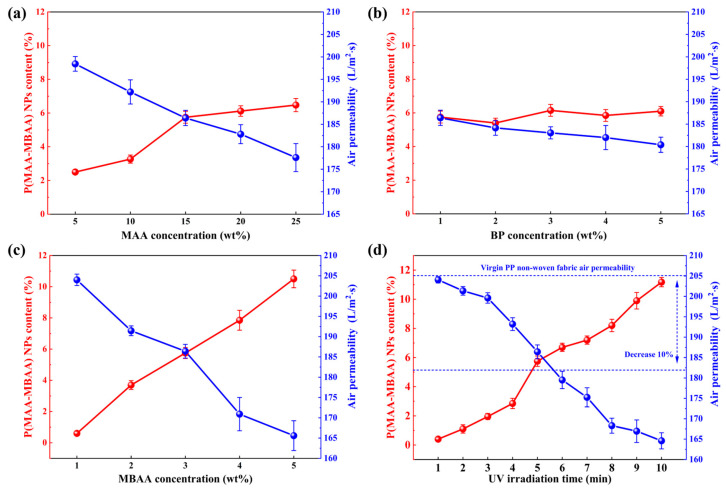
Factors affecting the P(MAA-MBAA) nanoparticle content in PP-P(MAA-MBAA) MNFs: (**a**) MAA concentration; (**b**) BP concentration; (**c**) MAA concentration; (**d**) UV irradiation time.

Figure 3 shows the effects of MAA concentration, BP concentration, MBAA concentration, and UV irradiation time on the P(MAA-MBAA) nanoparticle content in PP MNFs (Table S1). After the MAA concentration exceeded 15%, the P(MAA-MBAA) nanoparticle content increased slowly (Figure 3a), possibly due to the intensification of monomer self-polymerization under a high-concentration environment [40]. The increase in BP concentration showed little improvement in the content of P(MAA-MBAA) nanoparticles (Figure 3b), possibly due to the relatively short duration of UV irradiation, which was insufficient for generating a significant number of free radicals. The increase in cross-linker concentration helps improve the P(MAA-MBAA) nanoparticle content in PP MNFs (Figure 3c). The cross-linker can form the chain segments in the cross-linking process, preventing the free radicals’ chain termination reaction and the monomers’ self-polymerization reaction [34].

UV irradiation time was an effective parameter for controlling the content of P(MAA-MBAA) nanoparticles. Figure 3d presents the effect of irradiation time between 1 min and 10 min on the P(MAA-MBAA) nanoparticle content in the PP-P(MAA-MBAA) MNFs. The extension of UV irradiation time resulted in a marked increase in the P(MAA-MBAA) nanoparticle content, from 0.5% at 1 min to 11.4% at 10 min. The extension of UV irradiation time leads to increasing initiator decomposition, which causes a higher concentration of free radicals. However, it should be noted that after the P(MAA-MBAA) nanoparticle content exceeds 10%, the surface of the nonwovens produces a white covering, and the softness worsens (Figure S3).

Due to the substantial influence of high P(MAA-MBAA) nanoparticle content on the performance of PP MNFs, further investigation was conducted to explore the relationship between nanoparticle content and the air permeability of PP MNFs. In Figure 3a,b, it was observed that an increase in the P(MAA-MBAA) nanoparticle content in PP MNFs was accompanied by a corresponding decrease in air permeability. This decrease was more pronounced with higher P(MAA-MBAA) nanoparticle content, which was mainly attributed to the presence of P(MAA-MBAA) nanoparticles in the PP MNFs that block the fiber pores, resulting in reduced air permeability. Especially in the group after 10 min of UV irradiation, SEM images (Figure S4) show significant nanoparticle agglomeration, resulting in a large area of gaps and voids blocked, which is also the direct cause of the PP MNFs showing the presence of a white covering and the deterioration of softness. To reduce the influence of P(MAA-MBAA) nanoparticles on the air permeability and performance of PP MNFs, in this work, the group with less than 10% permeability reduction and the group with P(MAA-MBAA) nanoparticle content higher than 5% were selected for follow-up experiments. According to the data in Figure 3, the suitable concentrations of each part of the finishing solution are 15% MAA, 1% BP, and 3% MBA, and the UV irradiation time should be 5 min.

After being treated with a chlorinated solution, the P(MAA-MBAA) nanoparticles on PP MNFs can be converted to N-halamine. Due to the abundance of N-halamine groups within the P(MAA-MBAA) nanoparticles and their widespread distribution throughout the PP MNFs, the PP-P(MAA-MBAA)-Cl MNFs demonstrate an exceptionally high level of active chlorine content, which can be observed directly from the color change during the titration process (Figure S5). To further optimize the chlorination conditions and characterize the reusability of PP-P(MAA-MBAA)-Cl MNFs, the effects of NaOCl concentration, chlorination time, and multiple cycles of chlorination were investigated.

**Figure 4:**
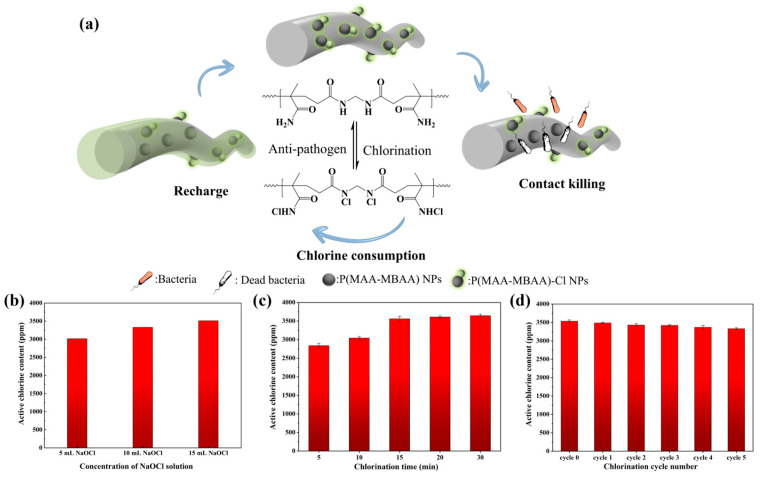
(**a**) Schematic illustration of antibacterial and chlorination processes of the PP-P(MAA-MBAA)-Cl MNFs. Effect of (**b**) concentration of NaOCl solution, (**c**) chlorination time, and (**d**) chlorination cycle number on active chlorine content in PP-P(MAA-MBAA)-Cl MNFs.

As shown in Figure 4b, the increase in NaOCl concentration promoted the growth of active chlorine content in PP-P(MAA-MBAA)-Cl MNFs in the same chlorination time. For example, with the NaOCl concentration increased from 5 mL to 15 mL, the active chlorine content increased from 3015 ppm to 3512 ppm. The main reason is that under the same pH conditions, the increase in NaOCl concentration is conducive to the rise of HOCl content in the solution, and the increased HOCl content is helpful for the conversion of N-halamine precursors to N-halamine [36]. However, in an acidic environment, when NaOCl concentration exceeded 15% in the chlorinated solution, this could easily lead to a large number of irritating gases separating from the solution. To stabilize the chlorinated solution’s concentration and avoid the pollution of the environment by irritating gases, the amount of NaOCl added in the subsequent chlorinated process was set to 15 mL. Moreover, the effect of chlorination time on active chlorine content in PP-P(MAA-MBAA) MNFs was also tested in this work, as demonstrated in Figure 4c. With the extension of chlorination time, the active chlorine content increases steadily. Still, between 15 min and 30 min of chlorination time, the rise of active chlorine content slowed down, indicating that the conversion of N-halamine gradually approached saturation during this period. Then, the rechargeability of the active chlorine of PP-P(MAA-MBAA)-Cl MNFs was evaluated in five cyclic tests. As shown in Figure 4d, after five repeated chlorine quenching/recharging cycles, the active chlorine content still exceeds 3300 ppm. The slight decrease in the active chlorine content could be attributed to the hydrolysis of the amide structures [23]. In general, PP-P(MAA-MBAA) MNFs still have high active chlorine content after five cyclic tests. Their rechargeability can effectively restore their protective effect and prolong their service life.

However, while the extremely high chlorine content in PP-P(MAA-MBAA)-Cl MNFs resulted in significant pathogen inactivation, it also resulted in an increased potential for toxicity. In mask applications, it is imperative to ensure the safety of PP-P(MAA-MBAA)-Cl MNFs. Studies have shown that when the active chlorine content in the material is 200 ppm, it can be used on the human body and has an excellent antibacterial effect [41]. The active chlorine content in PP-P(MAA-MBAA)-Cl MNFs can be achieved by controlling the chlorination process. In this experiment, based on the chlorination conditions of PP-P(MAA-MBAA)-Cl MNFs with an active chlorine content of 3512 ppm, the amount of NaOCl added to the chlorination solution was controlled at 5 mL, and the chlorination time was controlled at 5 min, resulting in a decrease in the active chlorine content in PP-P(MAA-MBAA)-Cl MNFs to the safety threshold of 200 ppm.

**Figure 5:**
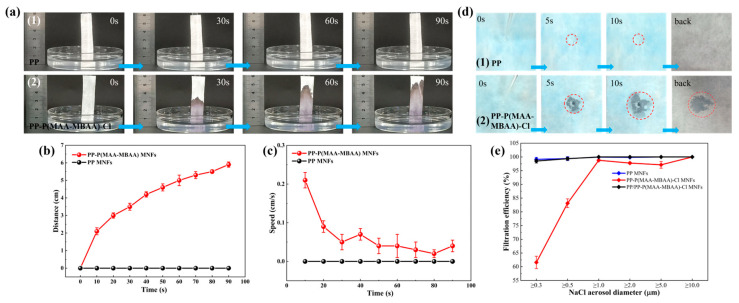
(**a**) Water transport performance of (**1**) PP and (**2**) PP-P(MAA-MBAA)-Cl MNFs at 0, 30, 60, and 90 s. Water transport curves of the PP and PP-P(MAA-MBAA)-Cl MNFs: (**b**) Distance–time curves during 90 s; (**c**) Speed–time curves during 90 s. (**d**) Unidirectional moisture conduction performance of (**1**) PP and (**2**) PP-P(MAA-MBAA)-Cl MNFs. (**e**) Filtration efficiency of PP, PP-P(MAA-MBAA)-Cl and PP/PP-P(MAA-MBAA)-Cl MNFs.

To assess the wearable comfort and protective effect of the PP-P(MAA-MBAA)-Cl MNF mask, we comprehensively measured the hydrophilicity, filtration efficiency, and antibacterial properties. Figure 5a presents the water transport performance of PP and PP-P(MAA-MBAA)-Cl MNFs. To visually assess the water transport capacity of the PP-P(MAA-MBAA)-Cl MNFs, a small amount of Kl and starch solutions were introduced into the water during testing. As shown in Figure 5(a1), no phenomenon occurred during the 90 s contact between PP MNFs and water. However, when PP-P(MAA-MBAA)-Cl MNFs came into contact with water, as shown in Figure 5(a2), the moisture continued to rise in the non-wetting direction of PP-P(MAA-MBAA)-Cl MNFs. Figure 5b,c show the distance–time curve and speed–time curve for nonwoven fabric absorbing moisture, respectively. After a test time of 90 s, the transmission distance of water on the PP-P(MAA-MBAA)-Cl MNFs is about 5.8 cm. In terms of the transmission speed of water in PP-P(MAA-MBAA)-Cl MNFs, water is transmitted quickly in the first 10 s and at a speed of about 0.05 cm/s in the last 80 s. Overall, PP-P(MAA-MBAA)-Cl MNFs can achieve rapid and continuous moisture absorption.

The above experiments show that the hydrophilic PP-P(MAA-MBAA)-Cl MNFs can directly absorb water in direct contact. However, as a functional layer, PP-P(MAA-MBAA)-Cl MNFs need to be added to the inside of the mask, and whether the hydrophilic PP-P(MAA-MBAA)-Cl MNFs can effectively absorb external moisture still needs to be verified. In the next test, PP-P(MAA-MBAA)-Cl MNFs were closely overlaid with PP spun-bonded nonwoven fabrics, and then water droplets were added to one side of the PP spun-bonded nonwoven fabric. In Figure 5(d1), due to the hydrophobic characteristics of the lower PP melt-blown nonwoven fabric, the water droplets added to the upper spun-bond nonwoven fabric cannot penetrate, and they remain on the upper layer, similar to a regular protective mask. When wearing a mask, if the water vapor exhaled by the human body cannot be promptly expelled, it will continuously accumulate and form droplets, causing the mask to adhere to the skin and reducing wearing comfort. However, when the lower layer consists of PP-P(MAA-MBAA)-Cl MNFs (Figure 5(d2), the water droplets can penetrate the upper layer and be fully absorbed, indicating that the mask with a PP-P(MAA-MBAA)-Cl MNF functional layer can effectively absorb external moisture and improve wearing comfort.

The excellent filtration efficiency of PP masks is crucial in preventing the human body from being infected by pathogens. In this respect, it is necessary to test whether the filtration performance of PP-P(MAA-MBAA)-Cl MNFs still meets the requirements after undergoing multiple solution treatments. Using a sodium chloride aerosol with diameters ranging from 0.3 to 10.0 μm as the medium, the filtration efficiency of 100 cm^2^ PP-P(MAA-MBAA)-CI MNFs was evaluated. The flow rate of the sodium chloride aerosol was set at 32 L/min. Filtration efficiency refers to the ratio of the weight or number of particles captured by the filter to the weight or number of particles contained in the air before filtration. Figure 5e illustrates the filtration efficiency of PP, PP-P(MAA-MBAA)-Cl, and PP/PP-P(MAA-MBAA)-Cl MNFs for sodium chloride aerosols of varying diameters. As depicted in Figure 5e, the filtration efficiency of PP-P(MAA-MBAA)-Cl MNFs notably decreases when filtering sodium chloride aerosols with diameters of ≥0.3 μm and ≥0.5 μm. This decrease can be primarily attributed to the loss of electrostatic charges in the polar nonwoven material, resulting from the solution treatment. Electrostatic attraction serves as the primary filtration mechanism in polar melt-blown polypropylene nonwovens. To address the inadequate filtration performance exhibited by PP-P(MAA-MBAA)-Cl nonwoven materials, a synergistic approach involving the combination of PP-P(MAA-MBAA)-Cl and PP MNFs was employed in this experiment. Comparatively, the filtration efficiency of PP/PP-P(MAA-MBAA)-Cl MNFs is significantly enhanced compared to PP-P(MAA-MBAA)-Cl MNFs alone. This combination approach holds great promise for improving the application of PP-P(MAA-MBAA)-Cl MNFs in protective face masks, such as in the design of the four-layer mask (Figure S6).

**Figure 6:**
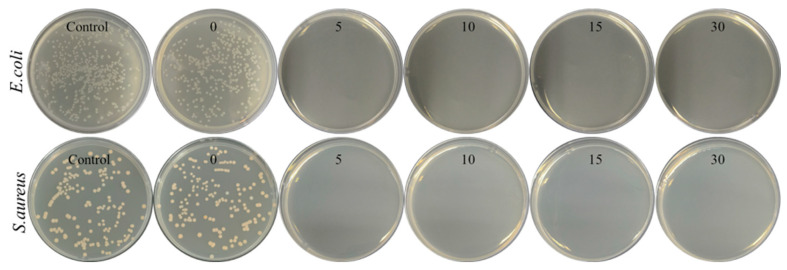
Antibacterial performance of the PP-P(MAA-MBAA)-Cl MNFs against *E. coli* and *S. aureus* under 5, 10, 15, and 30 min contact time.

The addition of antibacterial function can effectively inhibit the proliferation and spread of pathogens on the surface of the mask, reduce potential infection risks, and prevent the mask from becoming a carrier of pathogen transmission. Figure 6 illustrates the antibacterial performance of PP-P(MAA-MBAA)-Cl MNFs against *E. coli* and *S. aureus* at different contact times. As shown in Figure 6, no colonies (both *E. coli* and *S. aureus*) were observed on the agar plates after 5 min of contact with the PP-P(MAA-MBAA)-Cl MNFs. This antibacterial performance indicates that PP-P(MAA-MBAA)-Cl MNFs, with a high active chlorine content, exhibit a strong response to bacteria and demonstrate rapid bactericidal effects. In the same way, the antibacterial performance of PP-P(MAA-MBAA)-Cl MNFs with an active chlorine content of 200 ppm was also tested (as shown in Figure S7), also exhibiting excellent antibacterial effects. Therefore, incorporating PP-P(MAA-MBAA)-Cl MNFs into face masks holds great potential for enhancing their protective efficacy and reducing the risk of potential infections.

**Figure 7:**
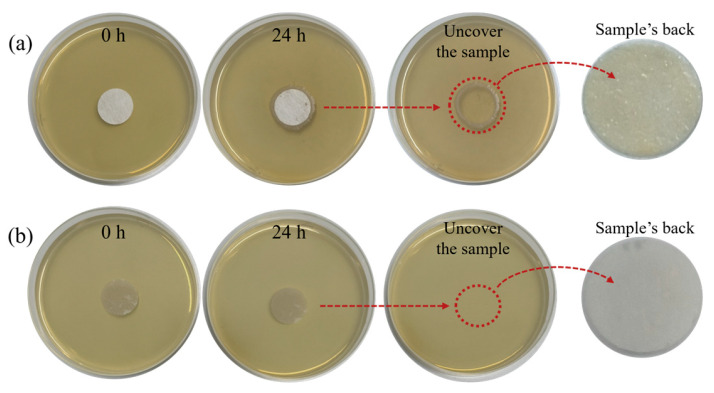
Aerosol antibacterial testing with (**a**) PP MNFs; (**b**) PP-P(MAA-MBAA)-Cl MNFs.

During the use of face masks, pathogens are often trapped in the form of aerosols or droplets. To further explore the application effectiveness of PP-P(MAA-MBAA)-Cl MNFs in real scenarios, the antibacterial effect of PP-P(MAA-MBAA)-Cl MNFs on aerosols containing bacteria was tested in this experiment. As shown in Figure 7a, bacteria trapped by PP MNFs grew around the PP MNFs on the agar plate. After the PP MNFs were uncovered, obvious *S. aureus* colony aggregates appeared on the back. However, in Figure 7b, no colonies formed by *S. aureus* were observed around or on the backside of the PP-P(MAA-MBAA)-Cl MNFs, indicating that the PP-P(MAA-MBAA)-Cl MNFs achieved the comprehensive disinfection of the bacteria in the aerosol. The antibacterial effect of PP-P(MAA-MBAA)-Cl MNFs with an active chlorine content of 200 ppm on aerosols containing bacteria was tested in the same manner. In the experimental results (as shown in Figure S8), neither agar plates nor the back of the samples exhibited any colonies, which shows that after setting the active chlorine to 200 ppm, PP-P(MAA-MBAA)-Cl MNFs can be safely used by people while still effectively eradicating bacteria within the aerosol.

## 4. Conclusions

In this study, a kind of PP MNF with high active chlorine content and super hydrophilicity was prepared by combining P(MAA-MBAA) nanoparticles. Under the triggering of benzophenone (BP), methacrylamide (MAA) and methylenebisacrylamide (MBAA) undergo a cross-linking reaction on the surface of PP MNFs and form numerous nanoparticles (P(MAA-MBAA) nanoparticles). The PP MNFs combined with P(MAA-MBAA) nanoparticles (PP-P(MAA-MBAA) MNFs) demonstrate notable additional characteristics, including superhydrophilicity, exceedingly high active chlorine content, and desirable chlorine reproducibility. Moreover, the PP-P(MAA-MBAA)-Cl MNFs display remarkable antibacterial activity after chlorination. Finally, this shows good application prospects for improving the protective effect of masks.

## Data Availability

The data presented in this study are available on request from the corresponding author.

## Supplementary Materials

The following supporting information can be downloaded at: https://www.mdpi.com/article/10.3390/polym15214335/s1.
