# Supplementary material for: N-Halamine-Based Polypropylene Melt-Blown Nonwoven Fabric with Superhydrophilicity and Antibacterial Properties for Face Masks
Source: Polymers (Basel). 2023 Nov 6;15(21):4335. doi: 10.3390/polym15214335 (PMC10648686; doi:10.3390/polym15214335)
Supplement: Supplementary file 1 [file polymers-15-04335-s001.zip › polymers-2647250-supplementary.pdf]

Supporting Information

Study on the Superhydrophilicity and Rapid Antibacterial Characteristics of Polypropylene Melt-blown Nonwoven Fabrics Functionalized with N-Halamine Nanoparticles

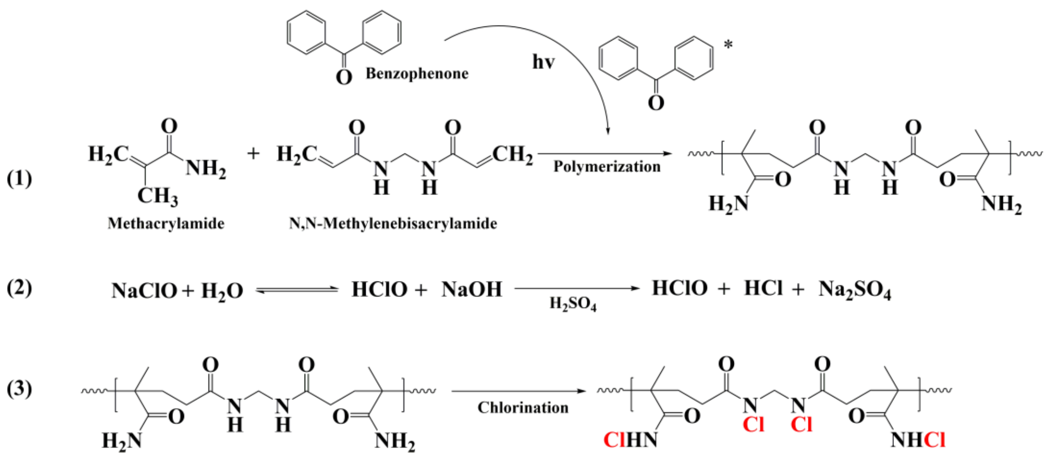

Figure S1. (1) Reaction process of P(MAA-MBAA) nanoparticles; (2) Chemical reactions of chlorinated solutions; (3) Reaction process of P(MAA-MBAA)-Cl

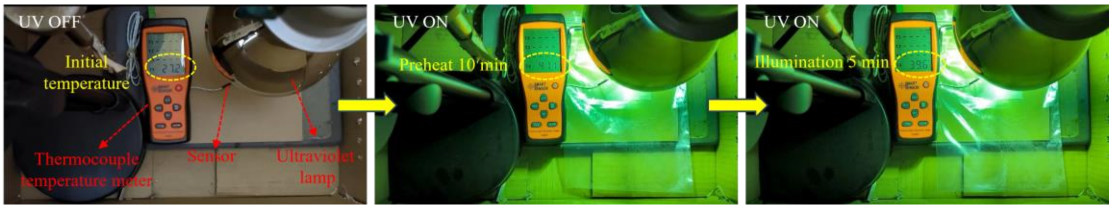

Figure S2. The optical picture of the experimental device and the process of UV illumination

Table S1. The effects of MAA concentration, BP concentration, MBAA concentration, and UV irradiation time on the P(MAA-MBAA) nanoparticle content and air permeability in PP MNFs

Variable / Number	MAA (wt%)	BP (wt%)	MBAA (wt%)	UV irradiation (min)	P-(MAA-MBAA) NPs content(%)	Air permeability ($L/m^2 \cdot s$)

1	5	1	3	5	2.5	198.4
2	10	1	3	5	3.2	192.2
3	15	1	3	5	5.7	186.4
4	20	1	3	5	6.1	182.8
5	25	1	3	5	6.4	177.6
6	15	1	3	5	5.7	186.4
7	15	2	3	5	5.4	184.1
8	15	3	3	5	6.1	183.0
9	15	4	3	5	5.8	182.0
10	15	5	3	5	6.1	180.4
11	15	1	1	5	0.6	204.0
12	15	1	2	5	3.7	191.4
13	15	1	3	5	5.7	186.4
14	15	1	4	5	7.8	170.9
15	15	1	5	5	10.5	165.6
16	15	1	3	1	0.4	204.0
17	15	1	3	2	1.1	201.3
18	15	1	3	3	1.9	199.6
19	15	1	3	4	2.8	193.2
20	15	1	3	5	5.7	186.4
21	15	1	3	6	6.7	179.5
22	15	1	3	7	7.2	175.2
23	15	1	3	8	8.2	168.3
24	15	1	3	9	9.9	166.9
25	15	1	3	10	11.1	164.6

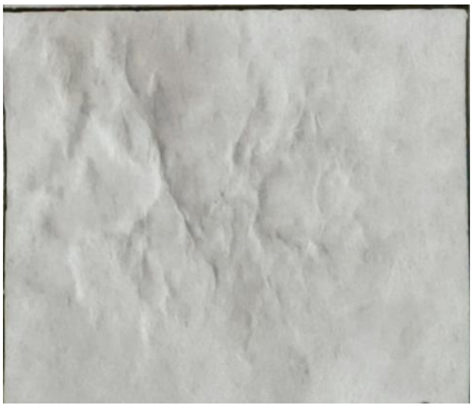

Figure S3. The optical picture of the PP-P(MAA-MBAA) MNFs that the P(MAA-MBAA) nanoparticles content exceeds 10%

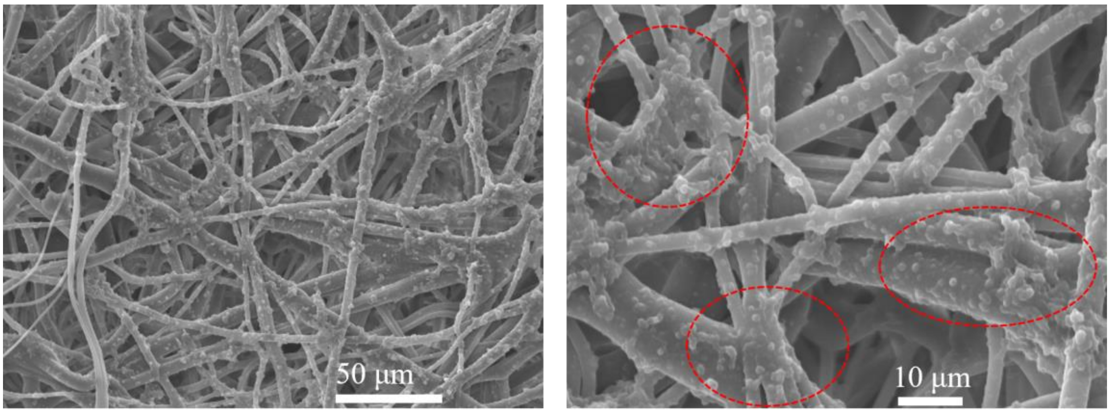

Figure S4. The SEM images of the PP-P(MAA-MBAA) MNFs after 10 minutes of UV illumination

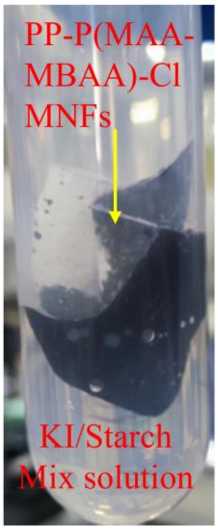

Figure S5. The optical picture of the PP-P(MAA-MBAA)-Cl MNFs reacting with the solution during titration

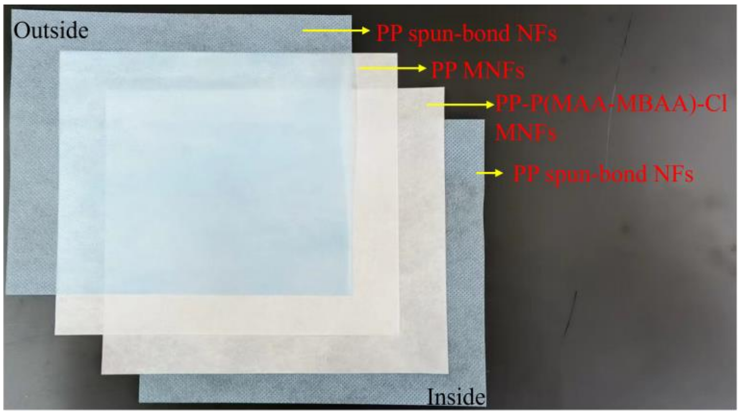

Figure S6. The design of the four-layer mask contained the PP-P(MAA-MBAA)-Cl MNFs

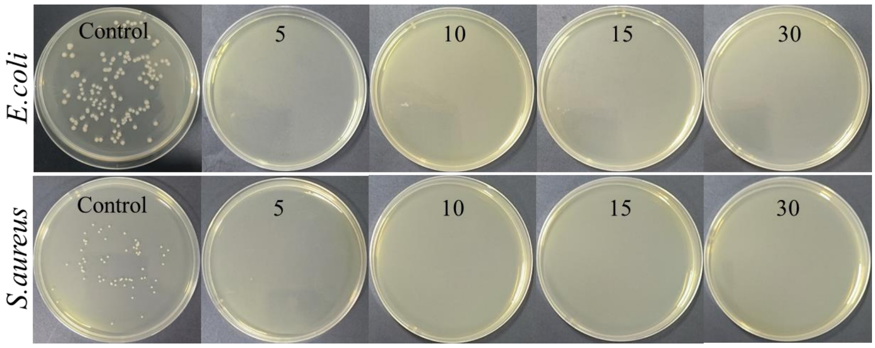

Figure S7. Antibacterial performance of the PP-P(MAA-MBAA)-Cl MNFs with an active chlorine content of 200 ppm against *E. coli* and *S. aureus* under 5, 10, 15, and 30 min contact time.

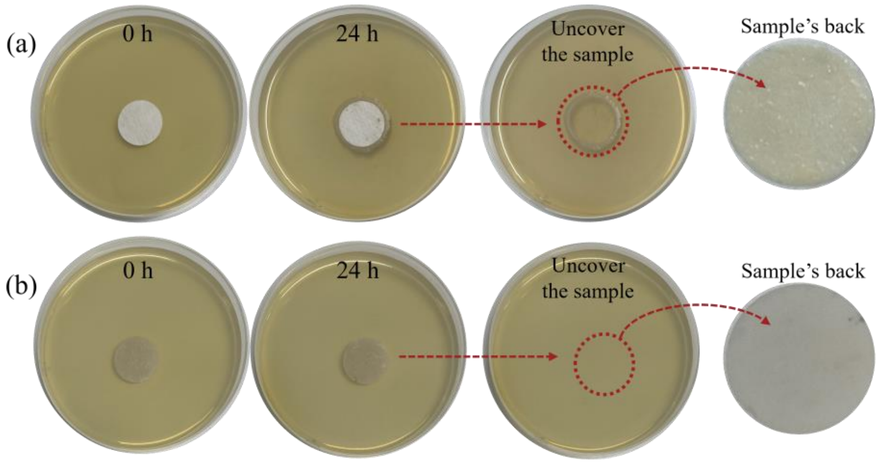

Figure S8. Aerosol antibacterial testing with (a) PP MNFs; (b) PP-P(MAA-MBAA)-Cl MNFs (200 ppm)
